# Reduced expression of CRH receptor type 1 in upper segment human myometrium during labour

**DOI:** 10.1186/1477-7827-7-43

**Published:** 2009-05-12

**Authors:** Binhai Cong, Lanmei Zhang, Lu Gao, Xin Ni

**Affiliations:** 1 Department of Physiology, Second Military Medical University, Shanghai 200433, PR China; 2 Department of Gynecology and Obstetric, Navy General Hospital, Beijing 10037, PR China

## Abstract

**Background:**

Corticotropin-releasing hormone (CRH) and CRH-related peptide are shown to modulate uterine contractility through two CRH receptor subtype, CRH-R1 and CRH-R2 during pregnancy. Through different signaling pathways, CRH-R1 maintains myometrial quiescence whereas CRH-R2 promotes smooth muscle contractility. We hypothesized that the expression of CRH receptors in myometrium might be changed during pregnancy and labour.

**Method:**

Immunohistochemistry, Western blot and RT-PCR were used to quantify the cellular localization, the protein levels and the mRNA variants of both CRH-R1 and CRH-R2 in upper segment (US) and lower segment (LS) myometrium from nonpregnant and pregnant women at term before or after labour.

**Results:**

CRH-R1 and CRH-R2 were predominately localized to myometrial smooth muscle cells in US and LS. The protein level of CRH-R1 in US was significantly down-regulated in pregnancy, with a further decrease at the onset of labour. However, the expression of CRH-R1 in LS remained unchanged during pregnancy and labour. No significant changes in CRH-R2 expression were observed in US or LS. Six variants of CRH-R1, CRH-R1alpha,-R1beta,-R1c, -R1e,-R1f and -R1g, were identified in nonpregnant and pregnant myometrium. CRH-R2alpha was identified in pregnant myometrium, whereas CRH-R2beta was identified in nonpregnant myometrium

**Conclusion:**

CRH-R1 and CRH-R2 are expressed in nonpregnant and pregnant US and LS myometrium. Changed expression of CRH receptors during labour may underlie the initiation of uterine contractility during parturition.

## Background

During pregnancy and labour the uterus undergoes dramatic changes in its contractile activity, which requires functional differentiation of the different regions of uterus. The upper segment (US) region of the uterus maintains a relaxatory phenotype throughout most of gestation to accommodate the growing fetus and adopts a contractile phenotype to cause expulsion of fetus at the onset of labour, whereas the lower segment (LS) must differentiate from a contractile phenotype into a relaxatory phenotype at labour, allowing passage of fetus [1]. It is known that abnormalities of this process have major clinical implications, such as preterm labour. However, to date the regulatory mechanisms for this process are poorly understood.

An increasing body of evidence suggests that corticotropin-releasing hormone (CRH) is an important factor in the regulation of human pregnancy and parturition [2-4]. During human pregnancy, the placenta and fetal membranes produce large amounts of CRH [5,6]. Synthesis of CRH in these tissues increases exponentially with advancing gestation and, by term, it is present in high concentrations in the maternal and fetal blood [6-8]. Abnormal increases or excessive levels of placental CRH are significant risk factors for an earlier onset of spontaneous birth [7,8]. Because of this, CRH has been proposed to regulate a placental clock that controls a cascade of physiological events leading to parturition [7]. Although the precise biological functions of CRH during pregnancy are not well defined, it appears to exert a number of effects in intrauterine tissues. It has been shown to stimulate prostaglandin production in placenta and fetal membranes [9-11], and to enhance estrogen production whilst reduce progesterone production in cultured placental trophoblasts [12,13]. A number of studies indicate that CRH is involved in the regulation of myometrial contractility during pregnancy [14,15].

It has been demonstrated that CRH belongs to a family of peptides that includes urocortin I (UCNI), urocortin II (UCNII) and urocortin III (UCNIII) as well as fish urotensin I and frog peptide sauvagine [16]. UCNI, UCNII and UCNIII have also been identified in human intrauterine tissues including placenta, fetal membrane and myometrium throughout gestation [15,17,18]. All the CRH family peptides exert their effects through two subtypes of CRH receptors, termed CRH-R1 and CRH-R2. These receptors shared 70% homology at amino acid level [19,20]. Several splice variants of the mRNA for CRH-R1 and CRH-R2 have been found, eight variants for CRH-R1 and three variants for CRH-R2 [20-22]. It has been shown that both CRH-R1 and CRH-R2 are expressed in human nonpregnant and pregnant myometrium [23-26]. Current evidence suggests that CRH-R1 and CRH-R2 activation exert distinct actions in the regulation of myometrial contractility during pregnancy. CRH-R1 activation up-regulates the expression of constitutive form of nitric oxide synthase, thereby promoting myometrium quiescence [27]. In contrast, CRH-R2 activation can activate ERK1/2 and RhoA pathways that actively promote myometrial contractility [28].

Our hypothesis is that differential expression of CRH-Rs may be important for regulating the contractile activity of uterus in the US and LS during pregnancy and labour. However, studies regarding CRH-R1 and CRH-R2 expression in human pregnant myometrium have so far been limited to LS region [23-26]. To date there is a lack of information about the expression of CRH-Rs within the US of human myometrium during pregnancy and labour.

The purpose of this study was to determine whether there were any changes in the expression of CRH-Rs in human US and LS myometrium during pregnancy and parturition. We therefore determined the protein levels of CRH-R1 and CRH-R2 in the US and LS by Western blot analysis and showed the localization of CRH-Rs in US and LS myometrium by immunohistochemistry. In addition, the mRNA splicing variants of CRH-R1 and CRH-R2 were also identified by RT-PCR.

## Methods

### Tissue collection

Paired upper and lower uterine segmental myometrial tissues from pregnant and nonpregnant women were collected in Navy General Hospital, the teaching hospital of Second Military Medical University, Beijing, China. Approval of this study was granted by human ethic committee of Navy General Hospital as well as human ethic committee of Second Military Medical University. Each patient signed approved informed consent form to participate in this study. Nonpregnant (NP) myometrium tissues were obtained from normal cycling patients (n = 8) undergoing hysterectomy for fibroids.

Pregnant myometrial tissues were collected at cesarean section from pregnant women at term gestation and were divided into two groups: (1) term no labour (TNL, 37–41 weeks, n = 10), (2) term labour (TL, 37–41 weeks, n = 9). Labour was defined as regular contractions (<5 min apart) plus membrane rupture and cervical dilation (>3 cm) with no augmentation. Indications for cesarean section included breech presentation, placenta previa, previous cesarean section, cephalopelvic disproportion, failure of labour to progress, fetal distress, or maternal request. None of the women included in this study had evidence of underlying disease (e.g. hypertension, diabetes, preeclampsia, intrauterine growth restriction, etc.). LS uterine samples were collected from the upper margin of the LS uterine incision, while US uterine samples were taken from just below fundus. The nonpregnant tissues were taken from the normal part of uterus without fibrosis contamination. Collected samples were placed in phosphate-buffered saline (PBS) on ice and transported to the laboratory. Myometrial tissues were separated from any serosal or decidual components and then rinsed in PBS, frozen immediately in liquid nitrogen and stored at -80 C for Western blot and PCR analysis. Parts of the tissues were also placed in 10% phosphate buffered formalin for immunohistochemical analysis.

### Immunohistochemistry

Paraffin sections (5 μm) were cut, rehydrated and microwaved in citric acid buffer to retrieve antigens. After inhibition of endogenous peroxidases with 3% H_2_O_2_, nonspecific antibody binding was blocked with 10% rabbit serum for 30 min. Serial sections were then incubated with specific antibodies against human CRH-R1 (sc-12381, Santa Cruz Biotechnology, Santa Cruz, California, USA) and CRH-R2 (sc-20550, Santa Cruz) (1:500) overnight at 4°C. The CRH-R1 antibody is directed against an epitope between amino acid positions 81 and 109 of CRH-R1 of human origin, where no sequence homology exists to CRH-R2. The CRH-R2 antibody was raised against a peptide mapping near the C-terminus of CRH-R2 of human origin. Mouse antihuman smooth muscle α-actin-specific monoclonal antibody (Dako Inc. Carpinteria, California) was used to stained the muscle cells. The bound antibodies were detected with the biotin-streptavidin-peroxidase system (UltraSensitive-SP-kit, MaiXin Biotechnology, Fuzhou, China) and diaminobenzidine (Sigma) was used as chromogen. Counterstaining was performed with hemalum. Negative controls were performed by substituting primary antibody with a normal serum or IgG in same dilution. To confirm the specificity of primary antibody, preabsorption of the primary antibody with a tenfold excess of the blocking peptides sc-12381P or sc-20550P (Santa Cruz) was performed.

### Western blot analysis

Approximately 70 mg of human myometrial tissue was homogenized in ice-cold lysis buffer consisting of 60 mM Tris-HCl, 2% sodium dodecyl sulfate (SDS), 10% sucrose, 2 mM phenylmethylsulfonyl fluoride (Merck, Darmstadt, Germany), 1 mM sodium orthovanadate (Sigma-Aldrich), 10 μg/ml aprotinin (Bayer, Leverkusen, Germany). Lysates were then quickly sonified in ice bath, boiled 5 min at 95 C, and stored at -80 C until used. Protein concentrations were measured using a modified Bradford assay and samples were diluted in sample buffer (250 mM Tris-HCl (pH 6.8), containing 4% SDS, 10% glycerol, 2% β-mercaptoethanol, and 0.002% bromophenol blue) and boiled for a further 5 min. Samples (50 μg) were separated on an SDS-8% polyacrylamide gel, and the proteins were electrophoretically transferred to a nitrocellulose membrane at 300 mA for 1.5 h in a transfer buffer containing 20 mM Tris, 150 mM glycine, and 20% methanol. The membrane was then blocked in TBS containing 0.1% Tween-20(TBST) and 5% dried milk powder (wt/vol) for 2 h at room temperature. After three washes with TBST, the nitrocellulose membranes were incubated with primary antibody for CRH-R1 or CRH-R2 (1:500) at 4°C overnight. After another three washes with TBST, the membranes were incubated with a secondary horseradish peroxidase-conjugated IgG (1:1000) for 1 h at room temperature and further washed for 30 min with TBST. Immunoreactive proteins were visualized using the enhanced chemiluminescence Western blotting detection system (Santa Cruz). The light-emitting bands were detected with X-ray film. The resulting band intensities were quantitated using an image scanning densitometer (Furi Technology, Shanghai, China). To control sampling errors, the ratio of band intensities to β-actin was obtained to quantify the relative protein expression level.

### Total RNA extraction and cDNA synthesis

Total RNA was extracted from the samples of myometrium by a method based on Chomczynski and Sacchi [29]. Briefly, frozen tissue samples were powdered under liquid nitrogen and homogenized in 1 ml of TRIzol reagent (Invitrogen, Grand Island, New York, USA). Subsequent extraction of total RNA from the tissues was conducted according to the protocol provided by the manufacturer. All RNA samples were treated with deoxyribonuclease I (Promega, Biotech. Co Ltd, Beijing, China). The purity and integrity of the RNA was checked spectroscopically and by gel electrophoresis. Two microgram of total RNA was reverse transcribed using the SuperScript^® ^first-strand synthesis system (Invitrogen) and stored at -20°C.

### PCR and nested PCR for CRH-R1 and -R2 variants

The specific primers for the amplification of the CRH-R2 variants and CRH-R1α/CRH-R1β were used as described previously [22,23,30]. The nucleotide sequences of the primers were as followings: (1) CRH-R2α, sense 5'-GAGCTGCTCTTGGACGGC-3', antisense 5'-GACAAGGGCGATGCGGTA-3'; (2) CRH-R2β, sense 5'-CCCTCACCAACCTCTCAGGTCC-3', antisense 5'-CAGGTCATACTTCCTCTGCTTGTC-3'; (3) CRH-R2γ, sense 5'-CTCAAGCAATCTGCCTACCT-3', antisense 5'-GGCTCACACTGTGAGTAGTT-3'; (4) CRHR1 α/β, sense 5'-GGCAGCTAGTGGTTCGGCC-3', antisense 5'-TCGCAGGCACCGGATGCTC-3'. PCR reaction solution consisted of 2.0 μl diluted cDNA, 0.4 μmol/L of each paired primers, 2.5 mmol/L Mg^2+^, 250 μmol/L deoxynucleotide triphosphates, 1 U Taq DNA polymerase (Qiagen, Beijing, China), and 1× PCR buffer. PCR reaction was set at 94 C (45 s), 58 C (45 s), 72 C (1 min) in a total 40–80 cycles with a final extension step at 72 C for 10 min.

Primers for nested PCR were previously described [21-23], and were designed to distinguish the different variants of CRH-R1. The nucleotide sequences of the first round primers were: exons 2–7, sense 5'-TCCGTCTCGTCAAGGCCCTTC-3', antisense 5'-GGCTCATGGTTAGCTGGACCAC-3'; exons 9–14, sense 5'-CCATTGGGAAGCTGTACTACGAC-3', antisense 5'-GCTTGATGCTGTGAAAGCTGACAC-3'. The nucleotide sequences of the second round primers were: exons 2–7, sense 5'-TGTCCCTGGCCAGCAACATCTC-3', antisense 5'-AGTGGATGATGTTTCGCAGGCAC-3';exons 9–14, sense 5'-GGGTGTACACCGACTACATCTAC-3', antisense 5'-TCTTCCGGATGGCAGAACGGAC-3'. RT-products (cDNA) from tissues were used as template for the fist round of PCR. After 40 cycles of amplification, 2 μL of the reaction mixture was used for additional 40-cycle amplification. The primers used in this second round of amplification were internal to the first set of primers.

Ten microliters of the reaction mixture were subsequently electrophoresed on a 1.5% agarose gel and visualized by ethidium bromide, using a 100 bp DNA ladder (Invitrogen) to estimate the band sizes. As a negative control for all of the reactions, distilled water was used in place of cDNA. The identity of the PCR products was confirmed by sequencing. Sequence data were analyzed using Blast nucleic acid database searches from the National Centre for Biotechnology Information (NCBI).

### Statistics

Protein expression levels of CRH-R1 and CRH-R2 were determined by densitometric analysis (Furi Technology, Shanghai, China). Peak count values were expressed as densitometric units. The data are presented as mean ± SEM. All data were tested for homogeneity of variance by Bartlett's test. The results indicated that the data were normally distributed. Student's *t*-test was used in the analysis of these data. One-way ANOVA with Student-Newman-Keuls was used for multiple comparisons. A *P *value < 0.05 was considered statistically significant.

## Results

### Localization of CRH-R1 and CRH-R2 in non-pregnant and pregnant human myometrium

**Figure 1:**
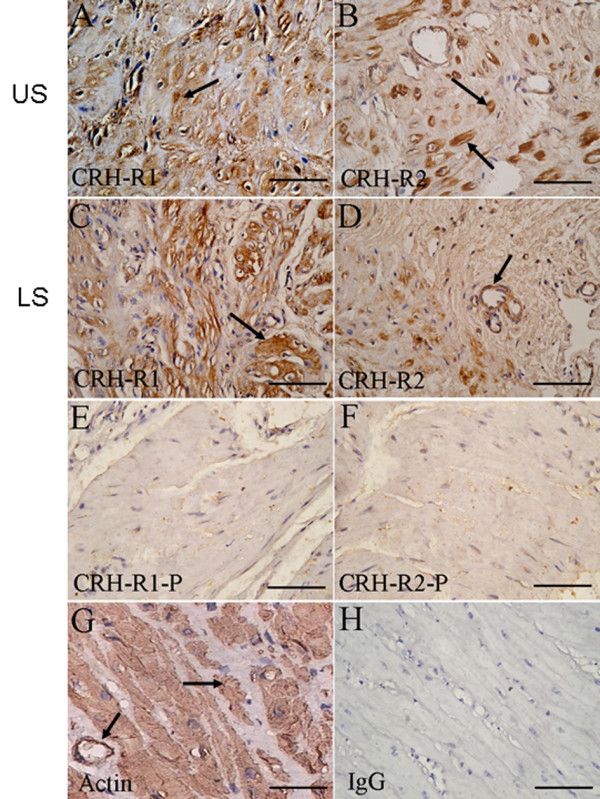
**Immunolocalisation of CRH-R1 and CRH-R2 in nonpregnant myometrium**. The figure shows representative sections of (A, B) US myometrium, and (C, D) LS myometrium. Immunostained with antibodies against (A, C) CRH-R1 and (B, D) CRH-R2. The primary antibody was either substituted with (E) CRH-R1 preabsorption antibody or (F) CRH-R2 preabsorption antibody. (G) Representative section immunostained with antibody against α-actin. (H) Negative control with normal mouse IgG. Original magnification × 400 (A-H). CRH-R1-P: CRH-R1 preabsorption. CRH-R2-P: CRH-R2 preabsorption.

**Figure 2:**
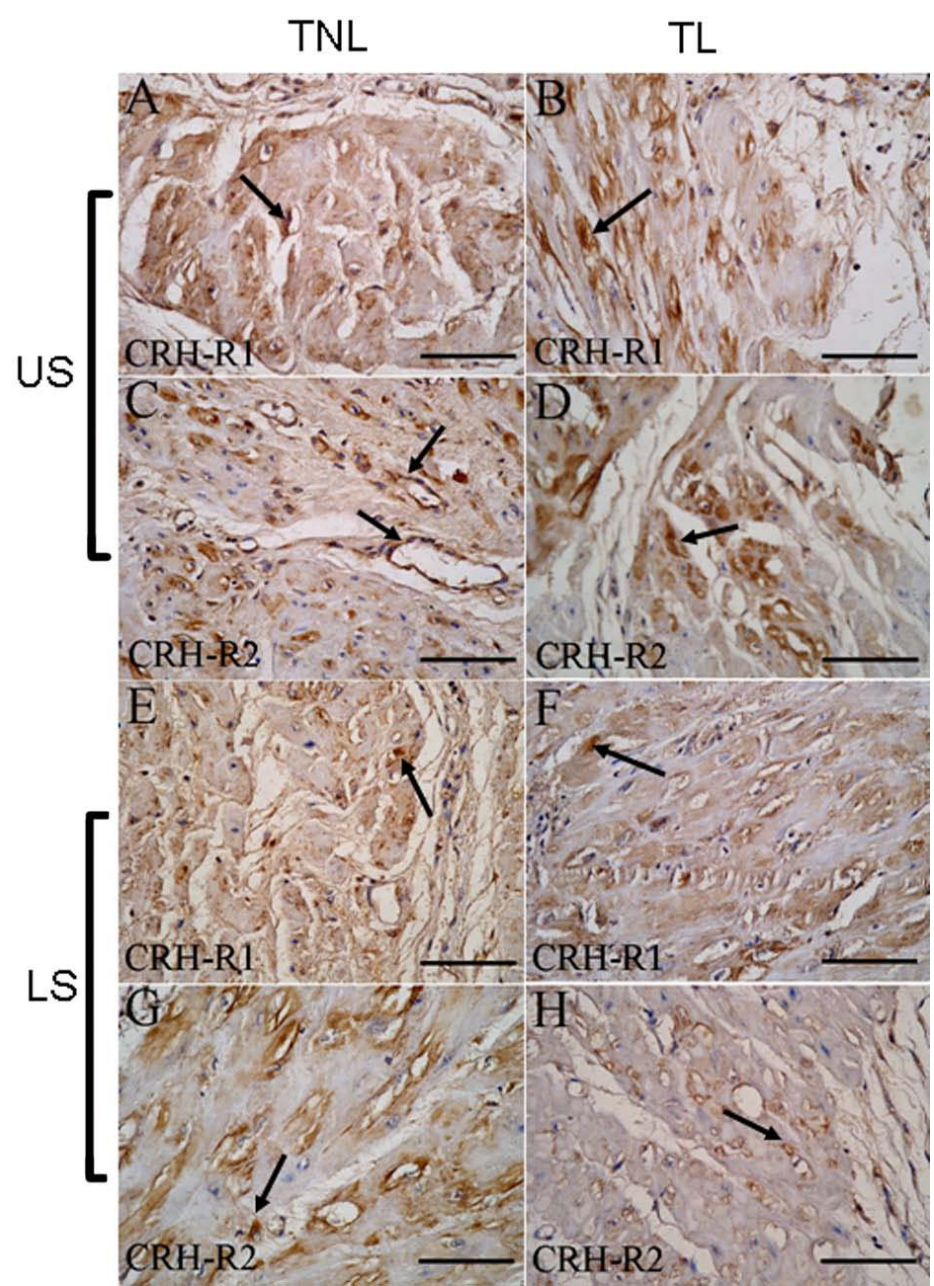
**Immunohistochemistry analysis of CRH receptors in myometrium from term labour (TL) and term nolabour (TNL) patients**. The figure shows representative sections of (A, C) US and (E, G) LS myometrium in TNL group, as well as (B, D) US and (F, H) LS myometrium in TL group. (A, B, E, F) Immunostained with CRH-R1 antibody. (C, D, G, H) Immunostained with CRH-R2 antibody. Original magnification × 400 (A-H).

Both CRH-R1 and CRH-R2 were identified in nonpregnant (Fig. 1) and pregnant (Fig. 2) myometrium. Localization of CRH-R1 and CRH-R2 showed that these receptors were highly expressed in the myometrial smooth muscle in US and LS (Fig. 1A–C and Fig. 2A, B and 2D–F arrowhead). Positive staining of CRH-R1 and CRH-R2 was also seen in vascular smooth muscle cells (Fig. 1D and Fig. 2C arrowheads). Staining in the US and LS was similar in each of groups and no dramatic changes in overall staining intensity or localization were observed with pregnancy or with labour (Fig. 2). CRH-R1 and CRH-R2 staining was eliminated when antibody was pre-absorbed by synthetic peptide (Fig. 1E and 1F, respectively).

### Pregnancy and labour associated with changes in the expression of CRH-R1 and CRH-R2

**Figure 3:**
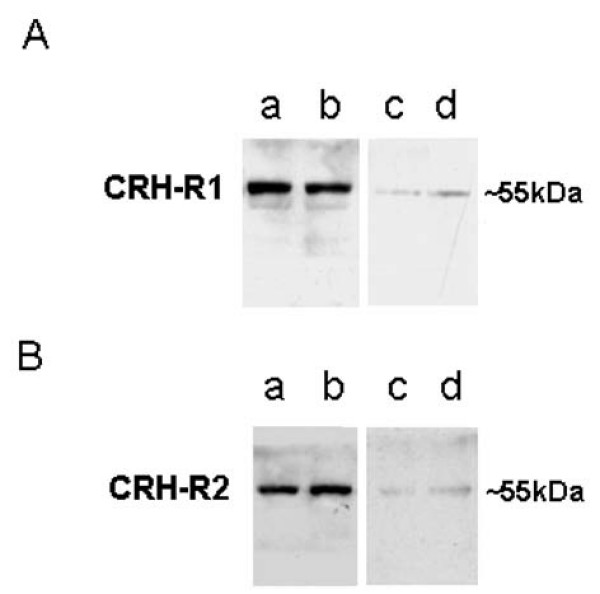
**Distribution of CRH-R1 and CRH-R2 in human myometrium by Western blot analysis**. (A) Western blot analysis of CRH-R1. a&b, immunoblotting using CRH-R1 antibody; c&d, preabsorption blots. (B) Western blot analysis of CRH-2. a&b, immunoblotting using CRH-R2 antibody; c&d, preabsorption blots.

As expected, Western blot analysis recognized a protein band of approximately 55 KDa corresponding to CRH-R1 in human myometrium (Fig 3A). A very faint band of about 47–50 KDa was also observed (Fig 3A). A single band of about 55 KDa corresponding to CRH-R2 was identified in human myometrium (Fig 3A). Preabsorption with corresponding peptides indicated the specificity of bands (c&d columns of Fig. 3A and 3B).

**Figure 4:**
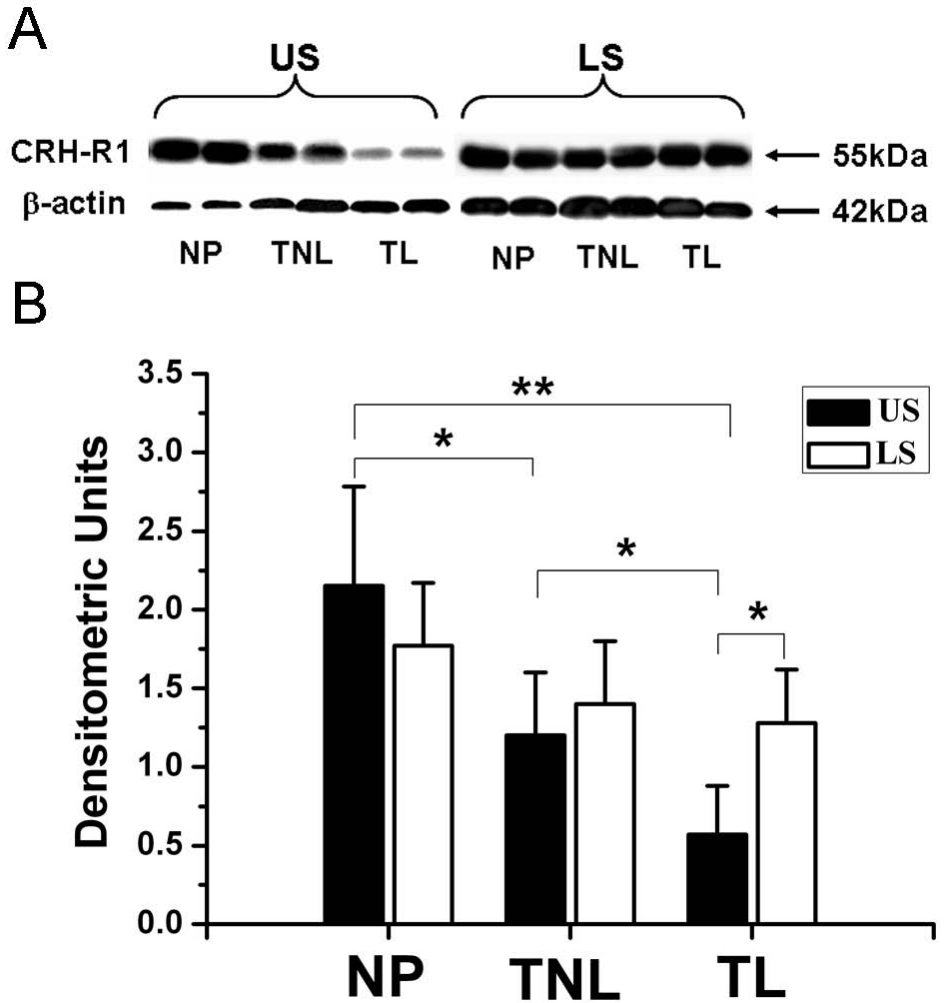
**Western blot analysis of CRH-R1 expression in nonpregnant and pregnant myometrium**. (A) Representative protein bands were presented. (B)The histogram shows the densitometric unit for each myometrium group. Data were expressed as mean ± SEM. *P<0.05, **P<0.01. NP, nonpregnant group (n = 8); TNL: term nolabour group (n = 10); TL: term labour group (n = 9).

With US myometrium, the protein level of CRH-R1 was down-regulated in pregnancy (NP versus TNL, *P *<0.05, Fig 4). It was further decreased at the time of labour (TL versus TNL, *P *< 0.05, Fig 4). With LS myometrium, although there was a trend of decreased CRH-R1 expression with pregnancy, the difference failed to reach significance (Fig 4, NP versus TNL, NP versus TL). No significant difference in CRH-R1 levels between TNL and TL group was observed.

**Figure 5:**
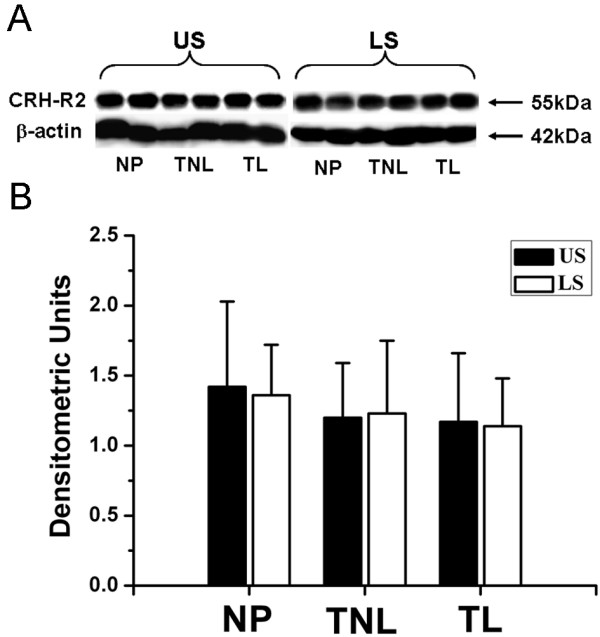
**Western blot analysis of CRH-R2 expression in nonpregnant and pregnant myometrium**. (A) Representative Western blot signals of CRH-R2 band in US and LS myometrium from nonpregnant and pregnant women. (B) The histogram shows the densitometric unit of CRH-R2 band for each group. Data were expressed as mean ± SEM. NP, nonpregnant group (n = 8); TNL: term nolabour group (n = 10); TL: term labour group (n = 9).

For CRH-R2 levels, no significant changes in associated with pregnancy or labour were found in US and LS myometrium (Fig. 5).

There was no difference in CRH-R1 expression between US and LS myometrim in NP and TNL samples. Interestingly, in TL samples, the LS myometrium expressed significantly more CRH-R1 when compared with US, in part due to the significant reduction in CRH-R1 expression in the US at the labour onset (Fig 4). No significant difference in CRH-R2 expression between US and LS myometrim was found across all groups (Fig 5).

### CRH-R1 and CRH-R2 variants in nonpregnant and pregnant myometrium

**Figure 6:**
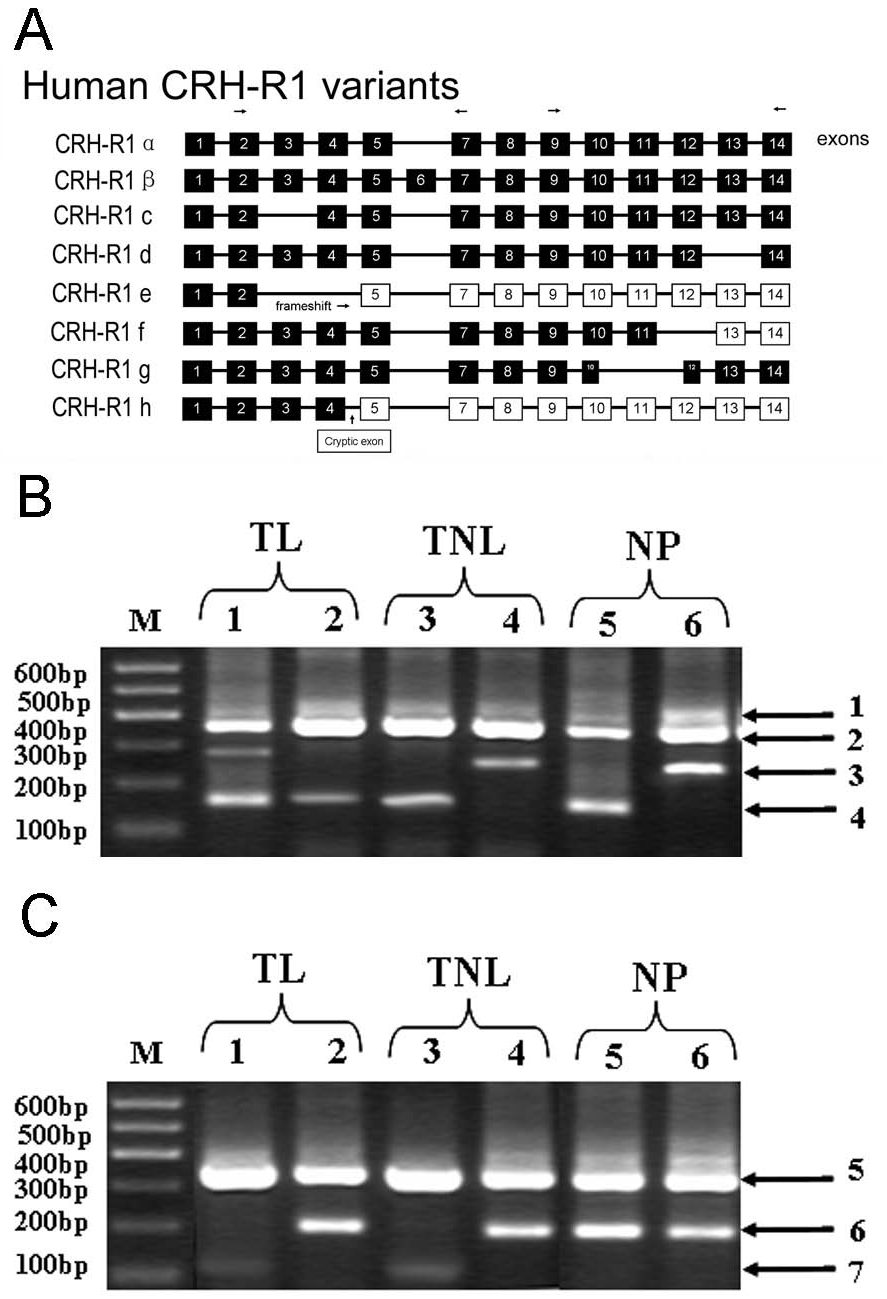
**PCR analysis of the human CRH-R1 variants in myometrium**. (A) CRH-R1 has eight different receptor isoforms (modification based on the scheme from ref 22). The representative results show the nested PCR products of CRH-R1in which (B) the primers that go across exon 2–7 were used, and (C) the primers that go across exon 9–14. Variants are distinguished by different molecular weight of amplified bands. Bands indicated by arrow 1 can only be CRH-R1β; arrow 2 can be CRH-R1α, -R1c, -R1f, or -R1g isoforms; arrow 3 are specific for isoform CRH-R1c; arrow 4 can be CRH-R1e. Bands indicated with arrow 5 can be CRH-R1α, -R1β, or -R1c isoforms; arrow 6 indicates CRH-R1f. arrow 7 indicates CRH-R1g. NP: nonpregnant group; TNL: term nolabour group; TL: term labour group; M: molecular weight marker. Lane 1,3,5: LS samples. Lane 2,4,6: US samples.

RT-PCR and nested PCR analysis showed that CRH-R1α,-R1β,-R1c, -R1e,-R1f and -R1g were identified in nonpregnant as well as pregnant US and LS samples (Fig 6B and 6C). Other CRH-R1 variants were undetected. CRH-R1α and -R1β were detected in all biopsies from pregnant and nonpregnant women, whereas CRH-R1c, -R1e,-R1f and -R1g were not detected in all biopsies. The detection rates of CRH-R1c, -R1e,-R1f and -R1g was various in NP, TNL and TL groups. For instance, within US, CRH-R1c was identified in 1/9TL and 3/10 TNL samples. CRH-R1e was detected in 2/9 TL and 3/10 TNL biopsies. CRH-R1f and -R1g were detected in all TL tissues and 4/10 TNL tissues. Within LS, 3/10 TNL and 4/9 TL tissues for CRH-R1c, 3/10 TNL and 5/9 TL tissues for CRH-R1e, 4/10 TNL and 6/9 TL samples for CRH-R1f, and 3/10 TNL and 5/9 TL tissues for CRH-R1g were identified.

**Figure 7:**
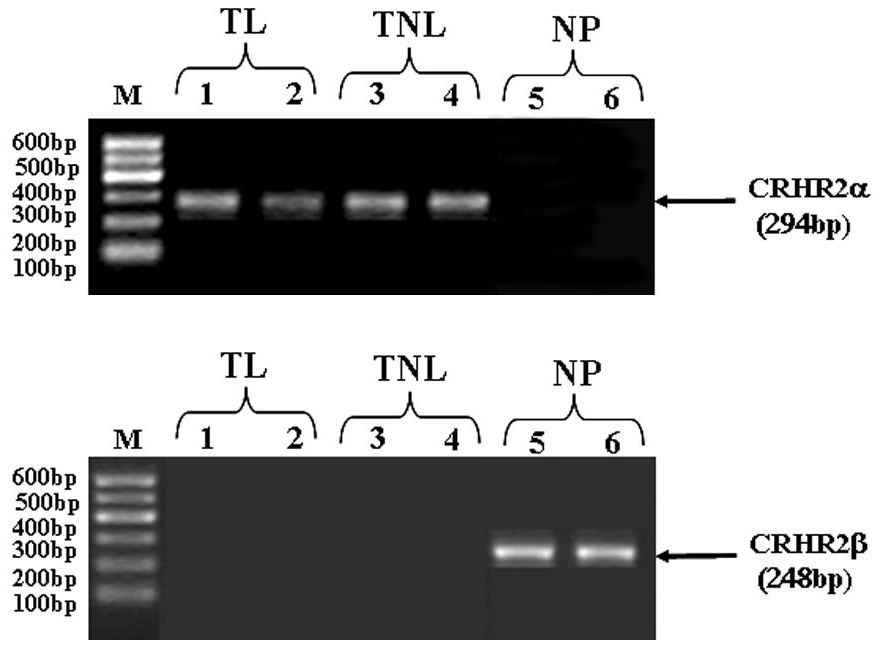
**PCR analysis of the human CRH-R2 variants in myometrium**. CRH-R2 variants were detected with specific primers. NP, nonpregnant group; TNL: term nolabour group; TL: term labour group; M: molecular weight marker. Lane 1,3,5: samples of US myometrium. Lane 2,4,6: samples of LS myometrium.

CRH-R2 has three variants, termed CRH-R2α, CRH-R2β and CRH-R2γ [20]. PCR analysis using specific primers for these CRH-R2 variants resulted in the detection of CRH-R2α in all of the pregnant myometrial biopsies and CRH-R2β in all of nonpregnant tissues (Fig 7). We are unable to detected CRH-R2γ (data not shown).

## Discussion

In the present study, we demonstrated that, for the first time, CRH-R1 and CRH-R2 expression in paired US and LS samples from pregnant women who were undergoing labour and not undergoing labour. We found that CRH-R1 is significantly reduced during labour, however, this decrease appeared to be restricted to the US region of uterus.

The presence of CRH-R1 in human myometrium is in accordance with published data [23-26,31]. However, the studies regarding the presence of CRH-R2 in pregnant myometrium are not consistent. Stevens et al [24] showed that CRH-R2 mRNA was detected in the myometrium of about 28% of the pregnant patients. In nonlabour myometria, CRH-R2 mRNA was undetectable. Wetzka and coworkers [25] also reported that expression of CRH-R2 mRNA was identified in 4 out of 6 pregnant myometrium biopsies. The present study as well as our previous study [23] showed that CRH-R2 mRNA and protein were identified in all specimens from pregnant and nonpregnant women investigated.

Some studies had shown pregnancy and labour associated changes in CRH-Rs expression in LS myometrium [24,31]. However, these studies only determined the mRNA levels of CRH-Rs. We previously showed that mRNA and protein levels of either CRH-R1 or CRH-R2 were not significantly changed in LS myometrium with labour [23]. In present study, within LS myometrium, no significant changes in protein level of both CRH-R1 and CRH-R2 were observed at term labour. However, within the paired US samples, CRH-R1 but not CRH-R2 was significantly down-regulated during pregnancy and labour.

Increasing body of evidence implicated that CRH exerts dual roles in the regulation of myometrial contractility during pregnancy, it maintains quiescence during pregnancy and promotes contractility after onset of parturition [14,15,32]. Current data from cultured myometrial cells suggested that CRH-R1 and CRH-R2 mediate distinct effects on the pregnant myometrium. Activation of CRH-R1 may maintain myometrium quiescence [15,27,32] whereas CRH-R2 activation promotes myometrial contractility [15,28]. Thus, a significant decrease in CRH-R1 but not CRH-R2 expression at term labour may help to facilitate contraction of myometrium for delivery fetus. In addition, it has been implicated that, at the time of labor, the fundus (US) of the uterus differentiates into the highly contractile activity during labour whereas LS region maintains relatively quiescent state [1]. Our findings that expression of CRH-R1 was reduced in fundus but not in LS at the time of labour may support the concept above.

Both CRH-R1 and CRH-R2 exist in several mRNA variants as results of alternative gene splicing, with eight variants for CRH-R1 and three variants for CRH-R2 identified to date [20-22]. Studies of Hillhouse groups have demonstrated that four CRH-R1 and two CRH-R2 variants are identified in pregnant myometrium [33-35]. Our present studies identified more CRH-R1 variants in nonpregnant and pregnant myometrium. It seemed that there were no obvious differences in CRH-R1 variants identified in US and LS myometrium.

Based on sequence analysis of different CRH-R1 variants and *in vitro *transfection studies, it is suggested that CRH-R1α is the main functional CRH-R1 variant, whereas other CRH-R1 variants may have various defects in binding or signaling properties [20,21,34-37] or modify action of CRH-R1α [38,39]. Hillhouse and Grammatopoulos proposed that the different expression pattern of CRH-R1 variants in different stage of pregnancy may account for, in part, dual roles of CRH in the regulation of myometrial contractility during pregnancy [32,40]. In our present study, there were no obvious differences in the expression pattern of CRH-R1 variants between TL and TNL group.

To date it is difficult to identify the splicing variants of CRH-R1 at protein level due to the lack of available antibodies recognizing individual splicing variants. Slominski's group showed CRH-R1 with different molecular weight by Western blot analysis in skin cells and proposed that CRH-R1 proteins with different molecular weight are dependent on glycosilation level and may also be the sliced forms of CRH-R1 [21,38,41]. In the present study, we also identified a very faint band of about 47–50 kDa and can be eliminated when the antibody was preabsorbed with synthetic peptide. However, the characteristics of the band are required to be studied in the future.

## Conclusion

Both CRH-R1 and CRH-R2 were expressed in US and LS myometrium of nonpregnant and pregnant uterus. CRH-R1 but not CRH-R2 was significantly down-regulated in US myometrium at the time of labour. Six CRH-R1 variants were identified in nonpregnant and pregnant US and LS myometrium. Our findings, the expression of CRH-Rs in myometrium during pregnancy and labour, are in accordance with roles of CRH receptors in the regulation of uterine contractility.

## Competing interests

The authors declare that they have no competing interests.

## Authors' contributions

BC carried out Western blot analysis and part of PCR analysis. LZ recruited patients, organized the collection of tissues and carried out immunohistochemistry work. LG did nested PCR analysis. XN conceived of the study, and participated in its design and coordination. All authors read and approved the final manuscript.
